# In Vitro/Vivo Mechanisms of Antibacterial Peptide NZ2114 against *Staphylococcus pseudintermedius* and Its Biofilms

**DOI:** 10.3390/antibiotics13040341

**Published:** 2024-04-08

**Authors:** Shuang Zhang, Na Yang, Ruoyu Mao, Ya Hao, Da Teng, Jianhua Wang

**Affiliations:** 1 Gene Engineering Laboratory, Feed Research Institute, Chinese Academy of Agricultural Sciences, 12 Zhongguancun Nandajie St., Haidian District, Beijing 100081, China; 2 Innovative Team of Antimicrobial Peptides and Alternatives to Antibiotics, Feed Research Institute, Chinese Academy of Agricultural Sciences, Beijing 100081, China; 3 Key Laboratory of Feed Biotechnology, Ministry of Agriculture and Rural Affairs, Beijing 100081, China

**Keywords:** antimicrobial peptide NZ2114, *Staphylococcus pseudintermedius*, mechanism of action, biofilm, mouse pyoderma model

## Abstract

*Staphylococcus pseudintermedius* is an opportunistic pathogen commonly found in canines, and has garnered escalating interest due to its potential for zoonotic transmission and increasing antimicrobial resistance. However, the excessive use of antibiotics and the characteristic of *S. pseudintermedius* forming biofilms make treatment challenging. In this study, the in vivo and in vitro antimicrobial activity and mechanisms of action of NZ2114, a plectasin-derived peptide, against *S. pseudintermedius* were investigated. NZ2114 exhibited potent antibacterial activity towards *S. pseudintermedius* (minimum inhibitory concentration, MIC = 0.23 μM) with a lower probability of inducing drug-resistant mutations and efficient bactericidal action, which was superior to those of mopirucin (MIC = 0.25–0.5 μM) and lincomycin (MIC = 4.34–69.41 μM). The results of electron microscopy and flow cytometry showed that NZ2114 disrupted *S. pseudintermedius’* cell membrane, resulting in cellular content leakage, cytoplasmic membrane shrinkage, and, eventually, cell death. The intracellular ROS activity and Alamar Blue detection showed that NZ2114 interferes with intracellular metabolic processes. In addition, NZ2114 effectively inhibits biofilm formation, and confocal laser scanning microscopy further revealed its antibacterial and anti-biofilm activity (biofilm thickness reduced to 6.90–17.70 μm). The in vivo therapy of NZ2114 in a mouse pyoderma model showed that it was better than lincomycin in effectively decreasing the number of skin bacteria, alleviating histological damage, and reducing the skin damage area. These results demonstrated that NZ2114 may be a promising antibacterial candidate against *S. pseudintermedius* infections.

## 1. Introduction

*Staphylococcus pseudintermedius* is a canine commensal or opportunistic pathogen, primarily causing pyoderma, surgical wound infections, abscesses, otitis externa, urinary tract infections, and osteomyelitis [1,2,3]. Moreover, *S. pseudintermedius* has been identified as a potential causative agent of nosocomial infections in humans who have close contact with dogs, including soft tissue infections, sinusitis, and endocarditis [1,3]. The data for incidence rates of *S. pseudintermedius*-related skin lesions in various canine breeds investigated showed that *S. pseudintermedius* is the predominant pathogen of pyoderma in dogs, and the high level of antimicrobial resistance and biofilm-forming ability exist among *S. pseudintermedius* isolates. Over 90% of the isolates obtained from the skin and nostrils of dogs with pyoderma were *S. pseudintermedius* [4]. It has been estimated the prevalence of multidrug-resistant (MDR) *S. pseudintermedius* ranged from 15.6% to 17% in the USA in 2001–2005 up to 62% in 2019–2021 in dogs with pyoderma [5,6]. Gharajalar et al. indicated that 94.59% of *S. pseudintermedius* isolates were positive for biofilm production [3]. Biofilms provide protection for microorganisms against external environmental stress and promote horizontal gene transfer. Biofilm-related infections are challenging to treat because of the increasing antimicrobial tolerance exhibited by bacteria growing in biofilm compared to planktonic cells [7].

Currently, the primary therapeutic approach for canines with pyoderma includes systemic administration with antibiotics or the application of topical formulations such as gels, creams, and other antibacterial agents and products [8]. Antibiotics have performed a leading role in the treatment of *S. pseudintermedius*-infected canine pyoderma; however, treatment has become complicated by frequent antibiotic resistance [8]. The rise in the prevalence of staphylococcal antimicrobial resistance has been also linked to pyoderma in canines, and prolonged antibiotic treatment, which is often necessary for severe cases of pyoderma, more easily leads to the emergence of drug resistance [6]. The threat from the zoonotic potential and increasing antimicrobial resistance, coupled with restrictions on antimicrobial prescribing for pets in some countries, adds a new dimension of public health implications for canine pyoderma treatment [9]. Therefore, there exists an urgent need for novel and effective antimicrobials as viable alternatives to antibiotic drugs in the treatment of canine pyoderma.

Antimicrobial peptides (AMPs) have received increasing attention due to their wider range of activities, making them a promising class of small-molecule peptides. Antimicrobial peptides have antimicrobial and immunomodulatory effects on bacteria, fungi, viruses, and parasites [10,11,12,13,14]. They possess the advantages of antibiotics in disease therapy such as rapid bactericidal action and the ability to provide vaccines with specific targets in disease prevention, and avoid the disadvantages, such as high variation and resistance in pathogens and high residue in animals [15]. AMPs could synergistically complement vaccines and antibiotics, establishing an iron triangle to effectively block the spread of drug resistance for animal healthcare [15,16]. Plectasin, a fungal defensin, was isolated from *Pseudoplectania nigrella* and displayed potent activity against *Staphylococcus* spp. and *Streptococcus* spp. NZ2114, moreover, a derived peptide of plectasin, exhibited better antibacterial activity toward *S. aureus* with low hemolytic activity [17], and NZ2114 also effectively inhibited the biofilm-forming ability of *Staphylococcus dysgalactiae* and *S. aureus*, eradicating biofilm [15,18]. Therefore, NZ2114 is a promising antimicrobial that can inhibit and eradicate *S. pseudintermedius* and its biofilms, and the former effect was reported in early work [19]. In addition, the heterologous expression of NZ2114 was successfully established by a high-yield *Pichia pastoris* expression system [17], which is a step towards a cost-effective means of development. What is more important is that an AMP fermentation and purification platform system with an industrial scale of 20 m^3^ and 30 m^3^ was established in China in 2019 and 2021, respectively [15], which broke the bottleneck of AMP industrialization. However, no systemic studies in vitro and vivo have focused on the process details, mechanisms and mode of action of NZ2114 against *S. pseudintermedius* and biofilm so far except for our previous partial preliminary observation [19].

In this study, we aimed to elucidate the in vitro antibacterial activity and mechanisms of NZ2114 towards *S. pseudintermedius* and investigate its anti-biofilm activity. In addition, we evaluated the in vivo efficacy of NZ2114 treatment using a mouse pyoderma model induced by *S. pseudintermedius*.

## 2. Results

### 2.1. In Vitro Antibacterial Assay

#### 2.1.1. Minimal Inhibitory Concentration (MIC) and Minimum Bactericidal Concentration (MBC) Determination

NZ2114 exhibited potent antibacterial activity towards *S. pseudintermedius* (Table 1). The MIC values of NZ2114 against the clinical isolates *S. pseudintermedius* CGMCC 1.90005 and *S. pseudintermedius* CGMCC 1.90024 were 0.23 µM, which was superior to those of mupirocin (0.25–0.5 µM) and lincomycin (4.34–69.41 µM). NZ2114 also exhibited lower MBC values than those of mupirocin and lincomycin (Table 1). The results indicated that NZ2114 displayed better antibacterial activity towards *S. pseudintermedius* than mupirocin and lincomycin.

#### 2.1.2. Dose-Killing Curve Assays

**Figure 1:**
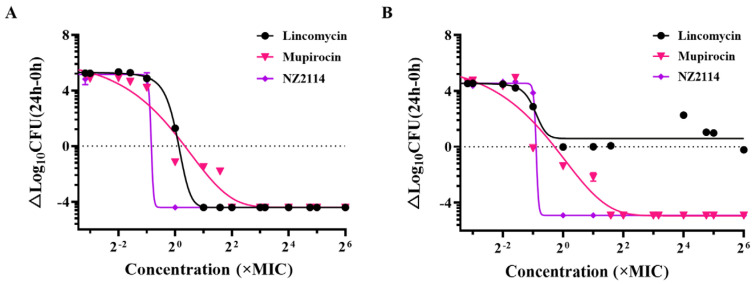
Dose−killing curves of NZ2114 against *S. pseudintermedius*. (**A**) *S. pseudintermedius* CGMCC 1.90024; (**B**) *S. pseudintermedius* CGMCC 1.90005.

The dose bactericidal curves of NZ2114 towards *S. pseudintermedius* are shown in Figure 1. NZ2114, mupirocin, and lincomycin all had significant dose-dependent effects on *S. pseudintermedius* CGMCC 1.90024. The actual maximum bactericidal effect of the three drugs reached Emax. The horizontal coordinate corresponding to the intersection point of the dashed line and the three bactericidal curves in the figure is the EC_50_ value, which indicates the concentration of the drug that caused the colony to reduce by half. Compared with lincomycin and mupirocin, NZ2114 exhibited a leftward shift in the curves and demonstrated a smaller EC_50_ value. The hillslope values of NZ2114, mupirocin, and lincomycin were −26.71, −0.30, and −2.49, respectively. For *S. pseudintermedius* CGMCC 1.90005, the actual maximum bactericidal effect of NZ2114 and mupirocin reached Emax, while that of lincomycin did not reach Emax and rebounded. The hillslope values of NZ2114, mupirocin, and lincomycin were −29.17, −0.425, and −5.967, respectively, and the EC_50_ value of NZ2114 was lower than those of mupirocin and lincomycin. The results indicated that NZ2114 exhibited superior bactericidal efficacy towards *S. pseudintermedius* compared with lincomycin and mupirocin, with a lower probability of inducing drug-resistant mutations.

#### 2.1.3. Bactericidal Effect Observation by Fluorescence Microscope

**Figure 2:**
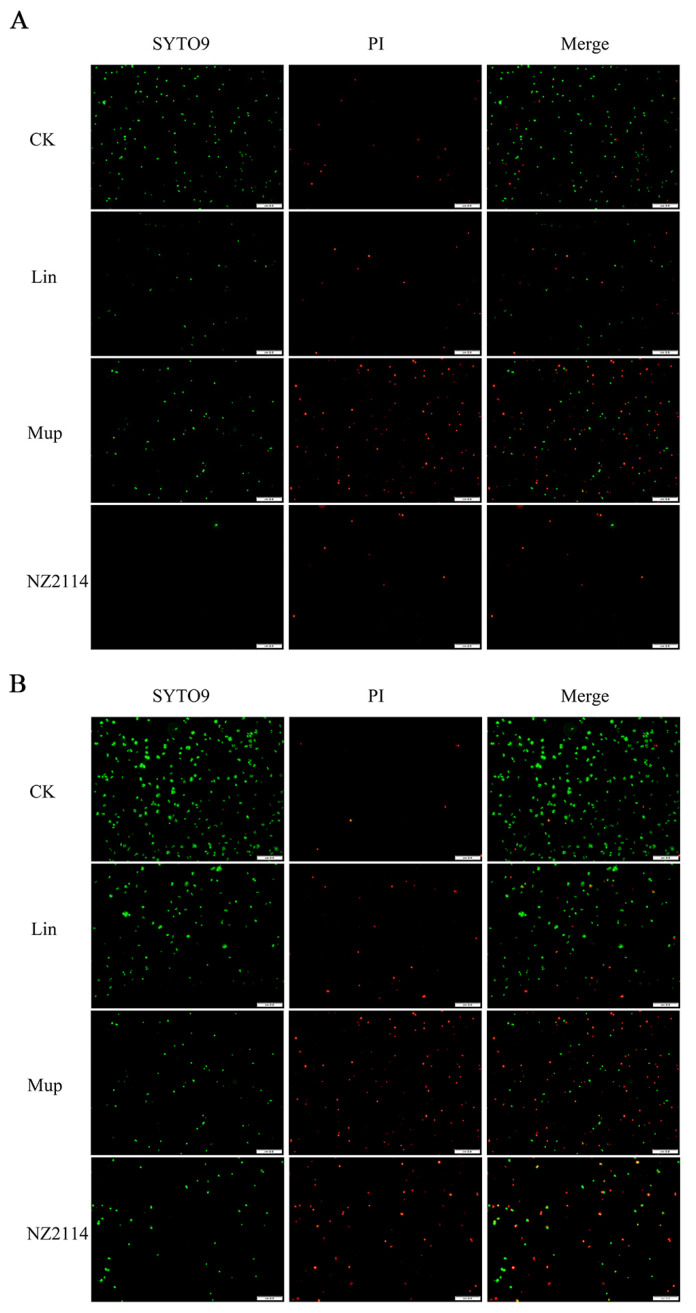
Bactericidal effect observation by fluorescence microscope. (**A**) *S. pseudintermedius* CGMCC 1.90024; (**B**) *S. pseudintermedius* CGMCC 1.90005. The *S. pseudintermedius* cells were treated with peptide or antibiotics at 4× MIC at 37 °C for 2 h. The cells were stained with SYTO 9 and PI. Fluorescence images of the same samples at 488 nm for SYTO 9 (green fluorescence; **left panels**), 561 nm for PI (red fluorescence; **middle panels**) and merged images (**right panels**) are shown. Lincomycin and mopirucin are abbreviated as “Lin” and “Mup”, respectively.

The bactericidal effect of NZ2114 on *S. pseudintermedius* was visually observed with propidium iodide (PI), SYTO 9 (green fluorescent nucleic acid stain), and red fluorescent nucleic acid stain using a fluorescence microscope (Figure 2). The treatment with 4× MIC NZ2114 led to a significant decrease in the bacterial number of *S. pseudintermedius* CGMCC 1.90024, indicating the superior bactericidal efficacy of NZ2114 compared to lincomycin and mupirocin (Figure 2A). For *S. pseudintermedius* CGMCC 1.90005, NZ2114 and mupirocin treatment caused a greater proportion of dead cells and higher cell membrane disruption compared with those of lincomycin. These findings further demonstrated the bactericidal activity of NZ2114 towards *S. pseudintermedius*. In addition, NZ2114 can destroy the cell membrane, facilitating the entry of PI dye into the cells.

### 2.2. Antibacterial Mechanism of NZ2114

#### 2.2.1. Effect of NZ2114 on Membrane Morphology and Cell Ultrastructure

**Figure 3:**
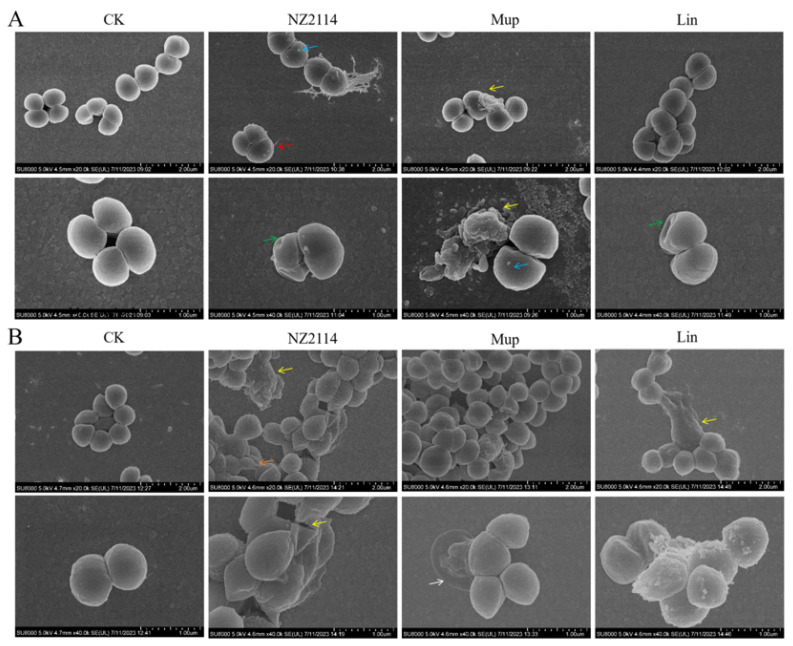
Scanning electron microscopy observation. (**A**) *S. pseudintermedius* CGMCC 1.90024; (**B**) *S. pseudintermedius* CGMCC 1.90005. The *S. pseudintermedius* cells were treated with 4× MIC NZ2114, mupirocin, or lincomycin for 120 min. Red arrows: cellular content leakage; green arrows: cell membrane shrinkage; yellow arrows: membrane breakage or cell rupture; blue arrows: cellular vesicles; white arrows: damaged cells; orange arrows: morphogenetic cells. Lincomycin and mopirucin are abbreviated as “Lin” and “Mup”, respectively.

**Figure 4:**
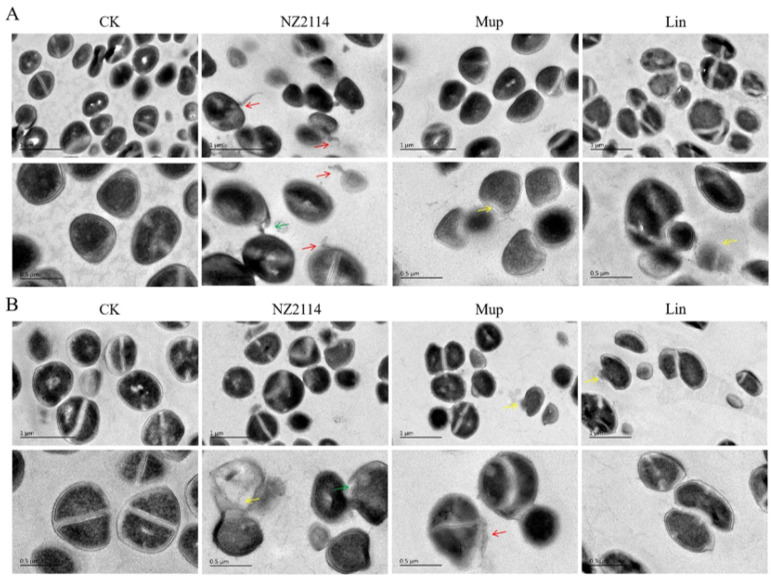
Transmission electron microscopy observation. (**A**) *S. pseudintermedius* CGMCC 1.90024; (**B**) *S. pseudintermedius* CGMCC 1.90005. The *S. pseudintermedius* cells were treated with 4× MIC NZ2114, mupirocin, or lincomycin for 120 min. Red arrows: cellular content leakage; yellow arrows: membrane breakage; green arrows: membrane delocalization. Lincomycin and mopirucin are abbreviated as “Lin” and “Mup”, respectively.

The impacts of NZ2114 on *S. pseudintermedius* cell morphology and integrity were visually observed using scanning electron microscopy (SEM) and transmission electron microscopy (TEM). As shown in Figure 3A, the untreated *S. pseudintermedius* CGMCC 1.90024 cells exhibited a smooth surface and intact cell morphology. After treatment with NZ2114, mupirocin, or lincomycin, the *S. pseudintermedius* cells exhibited cell damage such as vesicles on the cell surface (NZ2114 or mupirocin treatment), the shrinkage of cytoplasmic membranes (NZ2114 or lincomycin treatment), and intracellular content leakage (NZ2114 or mupirocin treatment). For *S. pseudintermedius* CGMCC 1.90005, after treatment with three drugs, the *S. pseudintermedius* cells exhibited varying degrees of damage (Figure 3B). NZ2114 treatment resulted in patches of cellular deformation, a tendency to membrane lysis, rupture of cell membranes, and the production of cellular debris. The mupirocin treatment caused cells to wrinkle and collapse. After treatment with lincomycin, pores appeared on the cell membrane, with vesicular protrusions on the surface (Figure 3B). The results of the TEM observation showed that after treatment with NZ2114, mupirocin, or lincomycin, *S. pseudintermedius* cells exhibited intracellular content leakage (NZ2114 and mupirocin), membrane delocalization (NZ2114), and membrane breakage (NZ2114, mupirocin, and lincomycin) (Figure 4).

#### 2.2.2. Membrane Integrity Analysis 

**Figure 5:**
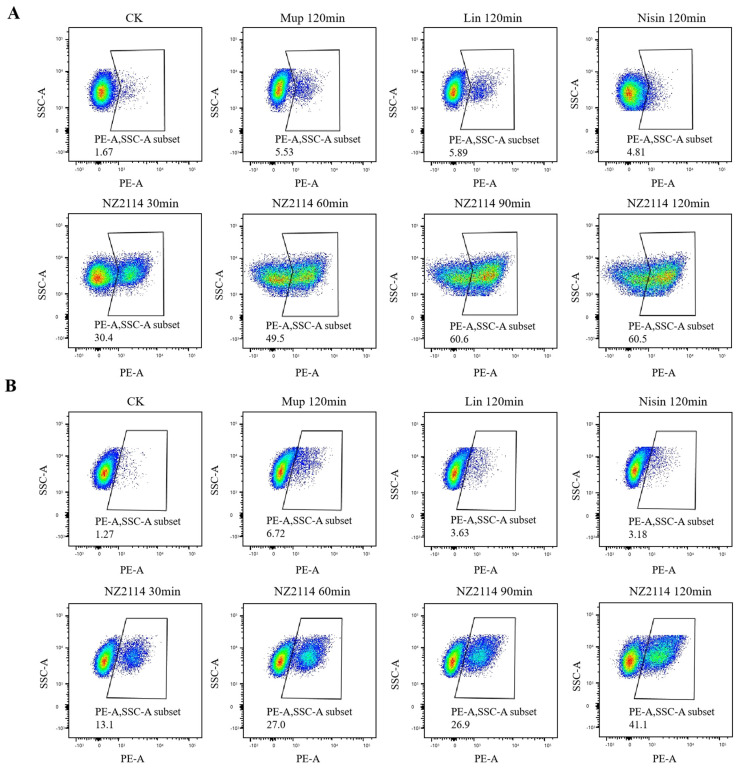
Membrane integrity analysis by flow cytometry. (**A**) *S. pseudintermedius* CGMCC 1.90024; (**B**) *S. pseudintermedius* CGMCC 1.90005. PI−stained *S. pseudintermedius* cells treated with 1× MIC NZ2114 for 30, 60, 90, and 120 min, respectively, were measured using a flow cytometer. Mup: treated with 1× MIC mupirocin for 120 min; Lin: treated with 1× MIC lincomycin for 120 min; Nisin: treated with 1× MIC nisin for 120 min; CK: cells not treated with peptide or antibiotics. Lincomycin and mopirucin are abbreviated as “Lin” and “Mup”, respectively.

In the untreated group, only 1.67% and 1.27% of *S. pseudintermedius* CGMCC 1.90024S and CGMCC 1.90005 cells were stained with PI, respectively, indicating that the membranes of *S. pseudintermedius* cells were intact (Figure 5). The percentages of PI-permeable *S. pseudintermedius* CGMCC 1.90024 cells after treatment with 1× MIC NZ2114 for 30, 60, 90, and 120 min were 30.4, 49.5, 60.6, and 60.5%, respectively (Figure 5A). For *S. pseudintermedius* CGMCC 1.90005, the PI-permeable percentages of cells after treatment with 1× MIC NZ2114 for 30, 60, 90, and 120 min were 13.1, 27.0, 26.9, and 41.1%, respectively (Figure 5B). The percentages of PI-permeable *S. pseudintermedius* cells after treatment with 1× MIC mupirocin, lincomycin, and nisin for 120 min did not exceed 6.8% (Figure 5). These data suggested that NZ2114 destroyed the cell membrane through its penetrating action.

#### 2.2.3. Super-Resolution Microscopy (SRM) Observation

**Figure 6:**
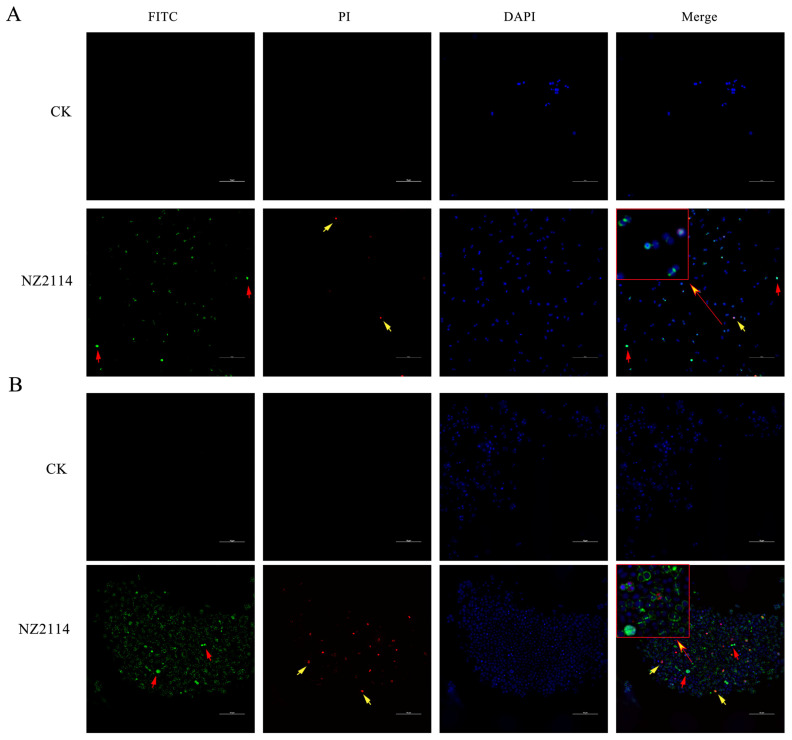
Super-resolution microscopy observation. (**A**) *S. pseudintermedius* CGMCC 1.90024; (**B**) *S. pseudintermedius* CGMCC 1.90005. The *S. pseudintermedius* cells were stained with SYTO 9 and PI. Intracellular DNA is shown in blue fluorescence (DAPI dye), whereas cells with a ruptured surface (altered membrane integrity) are shown in red fluorescence. Red arrows: FITC-NZ2114 entered into the cells; yellow arrows: PI dye entered into the cells.

The localization of FITC-labeled NZ2114 in *S. pseudintermedius* cells was detected using SRM to investigate their action targets against *S. pseudintermedius* CGMCC 1.90024S and CGMCC 1.90005. Only blue fluorescence (DAPI) was observed in the control group; however, red fluorescence (PI) was detected in the NZ2114 group (Figure 6), indicating that NZ2114 could increase cell membrane permeability, facilitating PI penetration and subsequent binding with nucleic acids. In addition, the majority of the green fluorescence (FITC-labeled NZ2114) was distributed around the cell surface, and a small portion entered into the intracellular region, suggesting that NZ2114 mainly acts on the cell membrane or wall.

#### 2.2.4. Calcein Leakage Assay

**Figure 7:**
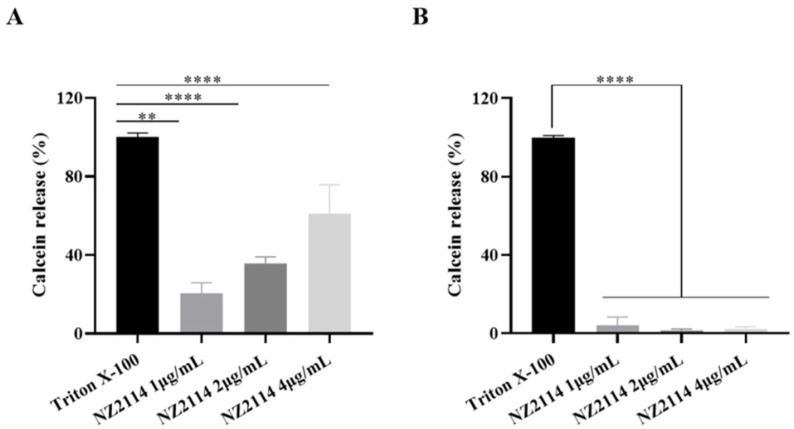
Calcein leakage assay. (**A**) Effect of NZ2114 on mimetic cell membranes of Gram-positive bacteria; (**B**) effects of NZ2114 on mimetic cell membranes of Gram-negative bacteria. All data were analyzed using one-way ANOVA and Bonferroni multiple comparison: ** *p* < 0.05; **** *p* < 0.0001.

To investigate the release of NZ2114-induced liposome leakage, the experiments were designed with two liposomes with different phospholipid ratios, respectively. The results demonstrated that NZ2114 exhibited a superior ability to disrupt the cell membranes of simulated Gram-positive bacteria; conversely, it had minimal disruptive effects on Gram-negative bacteria. At a 4 μg/mL concentration, NZ2114 induced the release rate of calcein up to 69.6% (Figure 7).

#### 2.2.5. Fluorescence Detection of Intracellular ROS Activity

**Figure 8:**
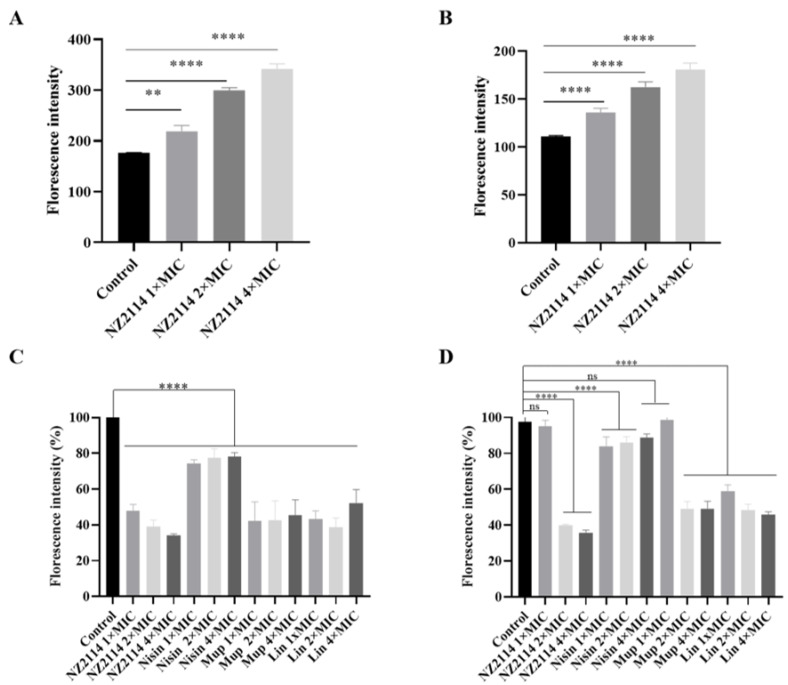
Effects of NZ2114 on cellular metabolism. (**A**,**B**) Effects of NZ2114 on intracellular reactive oxygen species levels; (**C**,**D**) Alamar Blue detection; (**A**,**C**) *S. pseudintermedius* CGMCC 1.90024; (**B**,**D**) *S. pseudintermedius* CGMCC 1.90005. Error bars represent means ± SEM, n = 3. ns, **, and **** represent insignificant, significant, and extremely significant, respectively (*p* > 0.05, *p* < 0.05, and *p* < 0.0001 by one-way ANOVA).

The intracellular ROS level was measured by the DCFH-DA fluorescence assay, in which non-fluorescent DCFH can be oxidized to a fluorescent DCF state in the presence of ROS enrichment. The fluorescence intensity in the NZ2114-treated group exhibited a dose-dependent increase (Figure 8A,B), demonstrating a positive correlation with ROS levels. Intracellular oxidative-stress-induced ROS can oxidize and impair bacterial cell membranes, implying that NZ2114 also intensifies membrane damage in a dose-dependent manner.

#### 2.2.6. Alamar Blue Detection of Cell Metabolic Activity

As shown in Figure 8C, against *S. pseudintermedius* CGMCC 1.90024, the peptide and antibiotics reduced intracellular fluorescence, and NZ2114 had the greatest effect on the cells, with a dose-dependent decrease in fluorescence with increasing concentrations of NZ2114. For *S. pseudintermedius* CGMCC 1.90005, the peptide and antibiotic treatments had an effect on the fluorescence of the cells (Figure 8D). Increasing concentrations of NZ2114 or lincomycin resulted in a dose-dependent reduction in the fluorescence of the cells: 2× MIC NZ2114 and mupirocin treatment significantly reduced the intracellular fluorescence intensity, and 4× MIC NZ2114 treatment showed the greatest effect on the cells. The results suggested that NZ2114 significantly altered the intracellular redox state and interfered with intracellular metabolic processes.

### 2.3. Effects of NZ2114 on Biofilm

#### 2.3.1. Inhibitory Effects of NZ2114 on Biofilm Formation

**Figure 9:**
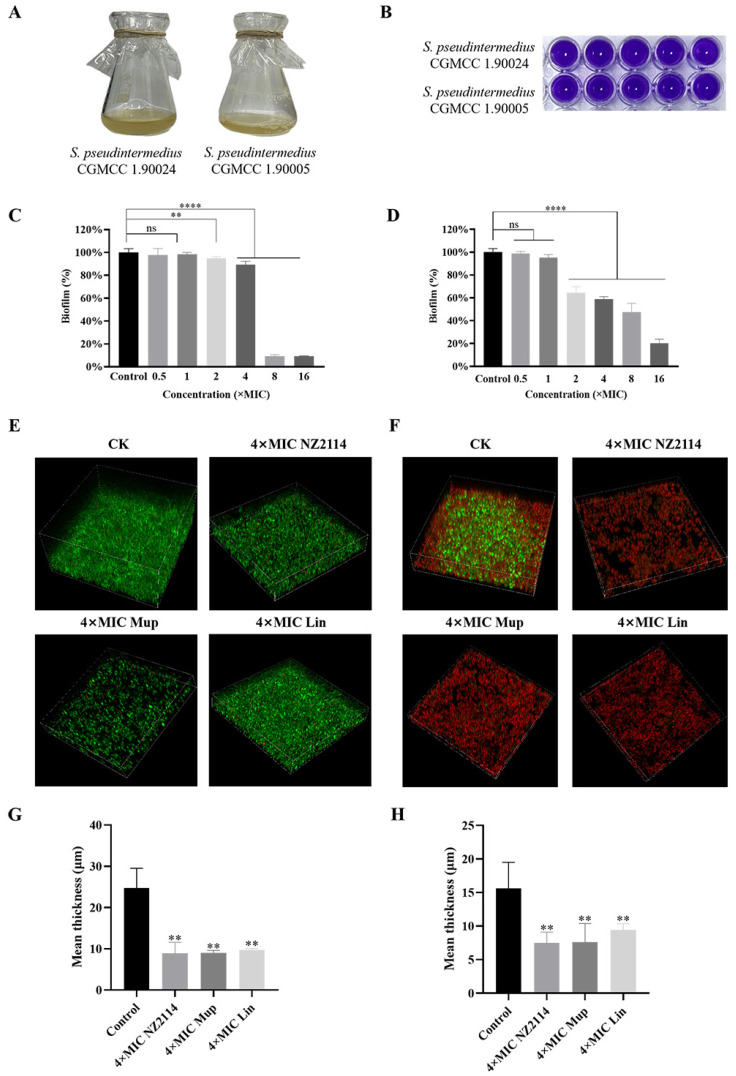
Effects of NZ2114 on biofilms. (**A**) Growth status of *S. pseudintermedius* strains; (**B**) biofilm-forming ability of *S. pseudintermedius* detected by violet staining; (**C**,**D**) inhibition effect of NZ2114 on biofilm formation; (**E**,**F**) biofilm observation by confocal laser scanning microscopy; (**G**,**H**) mean thickness of biofilm. The asterisk indicates a value statistically different from the control group; (**C**,**E**,**G**) *S. pseudintermedius* CGMCC 1.90024; (**D**,**F**,**H**) *S. pseudintermedius* CGMCC 1.90005. All experiments were independently repeated at least three times. Error bars represent means ± SEM, n = 3. ns, **, and **** represent insignificant, significant, and extremely significant, respectively (*p* > 0.05, *p* < 0.05, and *p* < 0.0001 by one-way ANOVA). Lincomycin and mopirucin are abbreviated as “Lin” and “Mup”, respectively.

The biofilm-forming ability of *S. pseudintermedius* CGMCC 1.90024 and CGMCC 1.90005 was assessed using crystal violet staining, revealing both strains of *S. pseudintermedius* to be highly proficient in forming biofilms (Figure 9B). As shown in Figure 9C,D, inhibitory effects of NZ2114 on *S. pseudintermedius* CGMCC 1.90005 biofilms were exhibited in a concentration-dependent manner and were obviously better than its inhibitory effects on *S. pseudintermedius* CGMCC 1.90024 biofilm at concentrations of 2× and 4× MIC. After treatment with NZ2114 at 16× MIC for 24 h, NZ2114 inhibited the formation of the initial biofilm of *S. pseudintermedius* CGMCC 1.90024 and CGMCC 1.90005 by 90.9% and 79.7%, respectively. This indicated that NZ2114 effectively inhibited *S. pseudintermedius* biofilm formation at an early stage.

#### 2.3.2. Biofilm Observation by Confocal Laser Scanning Microscopy (CLSM)

In order to further confirm the inhibition and eradication effect of NZ2114 on biofilm and internal bacteria, the treated *S. pseudintermedius* cells were observed by CLSM. As shown in Figure 9E,G, the untreated *S. pseudintermedius* CGMCC 1.90024 cells formed thick biofilms (24.70 ± 4.81 µM), and almost all cells were living cells (stained green). After treatment with NZ2114, mupirocin, and lincomycin, the thickness of the biofilm reached 8.90 ± 2.70, 9.00 ± 0.60, and 9.70 ± 0.35 µM, respectively. As shown in Figure 9F,H, the *S. pseudintermedius* CGMCC 1.90005 cells were found to form adhesive cells on the walls of the flask during culture, indicating a stronger biofilm-forming capacity. The untreated *S. pseudintermedius* CGMCC 1.90005 cells formed thick biofilms (15.60 ± 3.90 µM), and the dead cells at the bottom and edge showed red fluorescence. NZ2114, mupirocin, and lincomycin treatment effectively killed the living cells, and reduced the thickness of the biofilm to 7.50 ± 1.59, 7.60 ± 2.79, and 9.40 ± 0.97 µM, respectively. These results suggested that NZ2114 inhibited and eliminated *S. pseudintermedius* biofilm and its internal bacteria.

### 2.4. Efficacy of NZ2114 in Mice

**Figure 10:**
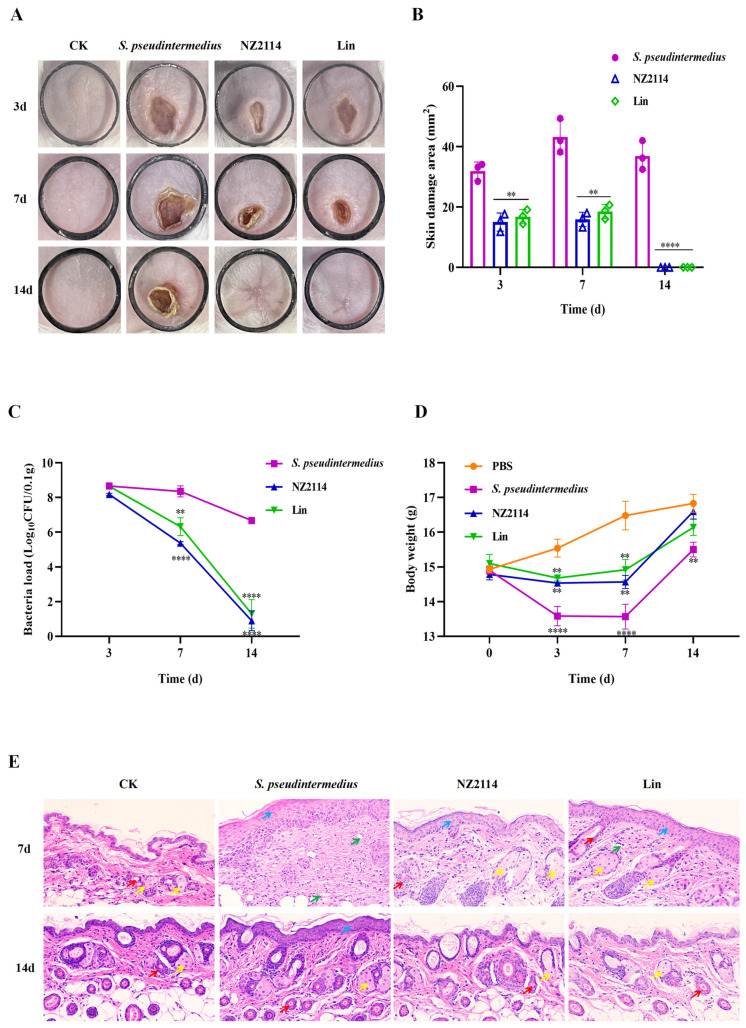
The therapeutic effect of NZ2114 in a mouse pyoderma model induced by *S. pseudintermedius* CGMCC 1.90024. (**A**) Photographs of abdominal wounds at days 3, 7, and 14 after challenge with *S. pseudintermedius* CGMCC 1.90024. The length and width of the black ring are 1.6 cm. (**B**) The wound area of different groups; results given as mean ± SEM (n = 3). (**C**) Effect of NZ2114 or lincomycin (Lin) on bacterial burdens in infected mouse skin. Data are presented as mean ± SEM (n = 6). (**D**) Body weight changes in different groups of mice. All data were analyzed by one-way ANOVA and Bonferroni multiple comparison: ** *p* < 0.05, **** *p* < 0.0001. (**E**) Histological assays of skin tissue from mice (magnification, ×200) at 7 and 14 d. CK: uninfected mice; *S. pseudintermedius*: infected mice without treatment; NZ2114: NZ2114-treated mice; Lin: lincomycin-treated mice. Red arrows: hair follicles; yellow arrows: sebaceous glands; blue arrows: thickening of the stratum spinosum in the epidermis; green clippings: inflammatory cell infiltration.

To test the in vivo efficacy of NZ2114, the abdomens of mice were infected with *S. pseudintermedius* CGMCC 1.90024 and treated with lincomycin and NZ2114. Thirty-six hours after the bacterial attack, the surface of the skin of the mice blanched, followed by redness and ulceration of the external skin caused by the internal bacteria at 48 h, and eventually complete wound formation at 72 h. The abscess was observed during the treatment period of 14 d and the abscess area was measured at 3, 7, and 14 d, respectively. Both NZ2114 and lincomycin treatment significantly alleviated the symptoms of the abscess and reduced its scope during the healing period (Figure 10A). The average abscess areas of the mice treated with NZ2114 at 3, 7, and 14 d were 15.0, 15.9, and 0 mm^2^, respectively, and those of the mice treated with lincomycin were 16.8, 18.5, and 0 mm^2^, respectively, far smaller than those of the negative group (31.9, 43.2, and 36.9 mm^2^, respectively) (Figure 10B).

The results of the determination of bacterial burden in each abscess showed that the number of bacteria was markedly different in the mouse skin between the 7th and 14th day after NZ2114 and lincomycin treatment compared with the untreated group (Figure 10C). The skin bacterial number in the NZ2114 treatment group decreased by three and six orders of magnitude on days 7 and 14, respectively, and those in the lincomycin treatment group decreased by two and four orders of magnitude on the 7th and 14th days, respectively. 

The mice’s body weights were measured on days 0, 3, 7, and 14, respectively (Figure 10D). After the bacterial attack, the sluggish state and lower food intake of the mice significantly contributed to a decrease in body weight. However, one week after NZ2114 or lincomycin treatment, the body weight significantly increased among the mice, especially among those in the NZ2114 treatment group, whose body weight substantially increased and was close to that of the PBS control group after two weeks.

The mice in the untreated group after bacterial attack had abnormal skin tissue structure on the 7th day: the spiny layer in the field of view was obviously thickened, no skin accessory glands could be seen, and there were a large number of inflammatory cells infiltrating in tissues (Figure 10E). After treatment with NZ2114 or lincomycin for 7 d, the spiny layer in the field of view thinned out compared with negative control group, skin accessory glands could be observed, and there were slightly fewer inflammatory cells. After a 14-day treatment with NZ2114 or lincomycin, the skin tissue of the mice basically returned to its normal state, while the spinous layer of the negative control group was still thicker, indicating that both NZ2114 and lincomycin treatment accelerated the recovery of the mice’s skin.

## 3. Discussion

*S. pseudintermedius* is an opportunistic pathogen frequently isolated from canines, capable of transmission between dogs and humans, with escalating antimicrobial resistance that poses significant public health concerns [3]. The urgent development of novel drug therapies is imperative to address this predicament. Researchers have demonstrated the potent bactericidal efficacy of AMP against multidrug-resistant bacteria with no/low resistance [20,21]. AMPs, which serve as the drug source library and therapeutic arsenal for the research and development of new antimicrobials, are produced by a broad range of organisms including plants, animals, bacteria, and fungi. Over 3000 natural AMPs are collected in the current antimicrobial peptide database (https://aps.unmc.edu, accessed on 1 February 2024). They have been regarded as promising alternatives to antibiotics, and their research and development have become a hotspot since this new century. In this study, the antibacterial activity, anti-biofilm activity, in vitro mechanism, and efficacy in mice of NZ2114 against *S. pseudintermedius* were investigated.

In previous studies, NZ2114 displayed bactericidal activity with high efficiency towards Gram-positive bacteria, including *S. suis*, *S. aureus*, *S. dysgalactiae*, and *Clostridium perfringens* [15,21,22]. In this study, NZ2114 displayed more potent antibacterial activity towards *S. pseudintermedius* than those of mupirocin (MIC: 0.25–0.5 µM) and lincomycin (MIC: 4.34–69.41 µM) (Table 1) similar to previous observation [19]. Jarosiewicz et al. evaluated the antimicrobial activity of seven AMPs (aurein 1.2, CAMEL, citropin 1.1, protegrin-1, pexiganan, temporin A, and uperin 3.6) against fifty-three methicillin-sensitive *S. pseudintermedius* (MSSP) and seven methicillin-resistant *S. pseudintermedius* (MRSP); all tested peptides were active against all reference- and clinical strains, in which uperin 3.6 showed the better antimicrobial activity (MIC_90_ = 2 μg/mL) [20]. In the dose-killing curves of *S. pseudintermedius*, NZ2114 exhibited a leftward shift in the curves and demonstrated a lower EC_50_ and a larger Hill coefficient (slope of the pharmacodynamic curve) than those of mupirocin and lincomycin (Figure 1). The hillslope is a slope with a more negative value indicating a steeper slope (hillslope values of NZ2114 ≤ −26.71), indicating a narrower mutation box and the lower probability of bacteria developing drug-resistant mutations. The results of the antibacterial activity of NZ2114 and its dose-killing curves exhibited its potent antibacterial activity towards *S. pseudintermedius* with a lower probability of inducing drug-resistant mutations and efficient bactericidal action. In our previous study, NZ2114 exhibited low hemolytic activity (less than 0.1%) on human erythrocytes at 128 μg/mL [17]. Given its low cell toxicity, high bactericidal efficiency, and low/nonresistance, NZ2114 preliminarily demonstrated its potential use as an antimicrobial agent.

The bactericidal mechanisms of AMPs differ from those of traditional antibiotics [23,24,25]. Antibiotics play a role by disrupting essential functions related to microbial growth or survival, such as inhibiting bacterial protein synthesis or modifying enzyme activity to kill bacteria, while bacteria can counteract these attacks by changing a gene. Conversely, AMPs primarily target bacterial cell membranes and act on them, inducing increased membrane permeability to effectively penetrate and kill bacteria [23,26,27]. To combat the AMP attack, the bacteria would need to undergo significant genetic modifications in order to alter the structure of their membrane, which is nearly impossible for bacteria to achieve. Therefore, the use of AMPs effectively reduces the possibility of resistance emergence [28,29]. Plectasin-derived peptide NZ2114 and MP1102 exhibit the ability to destroy the Gram-positive bacteria membrane, such as *C. perfringens*, *S. suis*, and *S. dysgalactiae* [15,21,22]. This study provides in vitro evidence of NZ2114’s mode of action on the *S. pseudintermedius* cell membrane. The effects of NZ2114 on the cell membrane of *S. pseudintermedius* were observed using SEM and TEM (Figure 3 and Figure 4), and the results revealed that NZ2114 caused significant changes in the morphology of *S. pseudintermedius*, obvious membrane surface shrinkage, and intercellular content leakage. The structure of numerous small vesicles occurs on the surface, and the production of membrane vesicles possesses the potential to induce an SOS response, thereby disrupting the membrane integrity [30,31,32]. The formation of membrane vesicles is a normal physiological process, usually intensified during biofilm formation or in response to stress. After *S. pseudintermedius* cells were treated with peptides, the cells were in a state of stress and the SOS response was induced inside the cells, causing cellular damage; vesicle formation is one of the external manifestations of this process. Through related studies, we can speculate that the phenomenon of “membrane delocalization” observed by TEM may be the result of the delocalization of membrane-binding proteins after reaction with peptides [33]. This also indicates that the site of action of the peptide on *S. pseudintermedius* may be on the membrane. This suspicion was confirmed by SRM assays, and the predominantly distributed peptides were observed around the membrane. Although the image shows a small amount of green fluorescence entering the cell, it is still uncertain whether the peptide can penetrate the membrane [34], but the damage caused by the peptide to the cell membrane is certain. In this experiment, the effect of peptide on the cell membrane was preliminarily detected in Figure 2, and the results were quantified by flow cytometry. It was observed that NZ2114 greatly increased the permeability of the membrane at 120 min, and the percentages of PI-stained *S. pseudintermedius* cells were 41.1–60.5% (Figure 5). The casein leakage induced by NZ2114 in PG/CL further demonstrated its damaging effect on cell membranes (Figure 7). Studies have shown that antimicrobial drugs can also interfere with intracellular metabolism while destroying membranes [35], and we examined the effect of peptides on intracellular metabolism through follow-up tests. ROS are important for cell growth, and elevated levels can lead to the inactivation of intracellular enzymes and cell membrane disruption, nuclear damage, and cell death. Previous studies have demonstrated that AMPs can result in cell death by enhancing the levels of ROS within fungi [36]; excessive levels of ROS can trigger a series of oxidative stresses, causing oxidative damage and inflammation in the body [37]. We evaluated the effect of NZ2114 on ROS, and observed that low concentrations of NZ2114 significantly increased ROS-induced cellular damage and the degree of intracellular auto-oxidation (Figure 8A,B). In addition, the detection results of cell metabolic activity by Alamar Blue indicated that NZ2114 could significantly alter the intracellular redox state and interfere with intracellular metabolic processes (Figure 8C,D). These findings of the intracellular ROS activity and Alamar Blue detection indicated that NZ2114 possesses the ability to disrupt cell metabolism and induce cell death. The in vitro antimicrobial mechanism of NZ2114 towards *S. pseudintermedius* is spatiotemporal. In the very beginning, there is a slight destruction of the cell membrane (Figure 3 and Figure 4). Then, NZ2114 penetrates into the cell and increases the levels of ROS within *S. pseudintermedius*(Figure 8A,B). Finally, severe damage to the membrane, such as cellular content leakage and even cell lysis, can result from this comprehensive effect. The dual mechanism involving both the destruction of the cell membrane and the interference of intracellular metabolic activities lays the foundation for the low drug resistance to NZ2114.

Biofilm formation is one of the prominent factors contributing to bacterial resistance. When bacterial cells attach to the surface and expand to the biofilms, they are embedded in an extracellular slimy polymeric substance like a shield, therefore becoming more resistant to antimicrobials than those of single or multiple bacteria [38,39]. In the clinical treatment of canines, *S. pseudintermedius* isolated from the skin of diseased dogs usually has a strong biofilm-forming ability [3,40,41]. AMPs typically exert their effects on biofilms through the following ways: (1) inhibiting biofilm formation in the aggregation and adhesion stages of microorganisms, such as the inhibition effect of HBD2 on *Pseudomonas aeruginosa*’s biofilm formation [42], and (2) the suppression of quorum sensing, such as the inhibitory effect of octopomycin on quorum sensing in *Acinetobacter baumannii* [43]. Biofilms with film-forming capacity are related to protein secretion and inflammatory response induction, and the degree of inflammation is positively correlated with the extent of film-forming [44,45]. NZ2114 has been found to play a role in inhibiting the biofilm of *S. dysgalactiae* and *S. aureus* [15,18]. Similarly, in this study, NZ2114 exhibited potent inhibitory and eradication effects on *S. pseudintermedius* biofilm. The inhibition rate of NZ2114 on the initial biofilm of *S. pseudintermedius* was 79.7–90.9% after treatment with 16× MIC for 24 h (Figure 9C,D). CLSM analysis further revealed that NZ2114 obviously decreased the thickness of *S. pseudintermedius* biofilm, eliminating it (Figure 9E,F).

To explore the in vivo efficacy of NZ2114, a mouse pyoderma model induced by *S. pseudintermedius* was established. The results of the abscess symptom observation showed that the treatment alleviated the symptoms and reduced the scope of the abscess (Figure 10A). In the NZ2114 treatment group, the average abscess areas of the mice at 3, 7, and 14 d were 15.0, 15.9, and 0 mm^2^, respectively, and those of the negative group were 31.9, 43.2, and 36.9 mm^2^, respectively (Figure 10B). The results of the determination of bacterial burden in the abscesses showed that the number of bacteria in the NZ2114 treatment group decreased by three and six orders of magnitude on the 7th and 14th days, respectively (Figure 10C). The rapid and efficient healing of skin wounds is crucial for safeguarding against infection sources while concomitantly properly restoring the skin function and structure layers. This is an extremely intricate and complex process [46]. The mice in the untreated group after bacterial attack had abnormal skin tissue structure on the 7th day: the spiny layer in the field of view was obviously thickened, no skin accessory glands could be seen, and there was a large quantity of inflammatory cells infiltrating the tissues (Figure 10E). After treatment with NZ2114 for 7 d, the thickness of the spiny layer had reduced, the skin accessory glands became visible, and there was a slight reduction in inflammatory cell infiltration. After a 14-day treatment with NZ2114, the skin tissue of the mice basically returned to its normal state, indicating that NZ2114 treatment could accelerate the recovery process of the mouse skin. In addition, other antibiotic alternatives derived from plant extracts, such as *Aloe vera* extract, volatile oil from *Atractylodis Rhizoma* (VOAR), and *Piper betle* leaf extract, also showed good therapeutic potential in staphylococcal pyoderma [47,48,49]. Dogs’ pyoderma treated with *A. vera* gel ointment had low haptoglobin and tumor necrosis factor-α concentrations than gentamicin [47]. VOAR can significantly reduce the skin bacterial load and has good therapeutic effect on mouse pyoderma induced by *S. pseudintermedius* [48]. Although these new treatment methods are still in the experimental stage, they have shown great potential, and can provide a pharmacological data reference for the development of new drugs for treating canine pyoderma in the future.

## 4. Materials and Methods

### 4.1. Strains, Mice, and Reagents

The clinical isolate *S. pseudintermedius* A2101 (CGMCC 1.90024) was obtained from Prof. Ding Mingxing’s laboratory at the School of Animal Medicine, Huazhong Agricultural University. *S. pseudintermedius* 19397 (CGMCC 1.90005) was obtained from China Agricultural University. These two pathogenic strains were stored at the China General Microbiological Culture Collection Center (CGMCC) and were utilized in the subsequent experimental studies. Six-week-old female BALB/c mice were purchased from Vital River Laboratories (Beijing, China), and were supplied with sterile feed and water and cultured in a sterile environment. The NZ2114 (GFGCNGPWNEDDLRCHNHCKSIKGYKGGYCAKGGFVCKCY) with purity >90% was prepared as per previous protocols [16]. Lincomycin hydrochloride was purchased from Meilun Biotechnology Company Limited. Mupirocin was purchased from Yuan Ye Biotechnology Co (Shanghai, China).

### 4.2. In Vitro Antibacterial Assay

#### 4.2.1. Determination of Antimicrobial Activity

The MIC values of the peptide and antibiotics were measured according to the microbroth dilution method [50]. In brief, 90 μL of the *S. pseudintermedius* cells in mid-log phase was diluted with MHB medium (BEIJING AOBOXING BIO-TECH Co., Ltd., Beijing, China) to 1 × 10^5^ CFU/mL and 10 μL of the diluted peptide or antibiotics with final concentration (1.25–1280 μg/mL) were added into a 96-well plate and incubated for 18–22 h at 37 °C. The MIC value was determined as the minimum concentration of peptide or antibiotics at which no bacterial growth was visible. The results were determined as the drug concentration corresponding to killing 99.9% of the bacteria as the MBC values of the antimicrobial drugs against *S. pseudintermedius*.

#### 4.2.2. Dose-Killing Curve Assays

The dose-killing curve assays were performed to assess the pharmacodynamics of NZ2114 against *S. pseudintermedius* [51]. The mid-log phase bacteria were diluted with MHB medium to 1 × 10^5^ CFU/mL. The cell suspension (180 μL) and diluted NZ2114 (20 μL) were added to a 96-well plate at the final NZ2114 concentrations of 0.0625-64× MIC, and co-cultured at 37 °C for 24 h. Subsequently, 100 μL of the sample was taken from the bacterial suspension at different points in time and plated on MHA medium for colony counting. Mupirocin and lincomycin were used as positive controls.

#### 4.2.3. Bactericidal Effect Observation by Fluorescence Microscope

The *S. pseudintermedius* cells in mid-log phase were diluted to 5 × 10^8^ cells/mL, and co-cultured with peptide or antibiotics at 4× MIC at 37 °C for 2 h. The cells were washed with PBS and PI (Sigma-Aldrich LLC., Shanghai, China), SYTO 9 (Beyotime Biotech. Inc, Shanghai, China), and the red fluorescent nucleic acid stain were added. The mixture was incubated for 15 min in the dark, and then washed with PBS. The 1 mL mixture was concentrated to 200 μL and 10 μL of the concentrated solution was drawn out and used to coat a glass slide. The status of the bacteria was observed using a 1000× fluorescence microscope (OLYMPUS DP73, Tokyo, Japan) [52].

### 4.3. Antibacterial Mechanism of NZ2114

#### 4.3.1. Electron Microscopy Observation

The mid-log phase *S. pseudintermedius* cells were treated with 4× MIC NZ2114 at 37 °C for 2 h and fixed with 2.5% glutaraldehyde at 4 °C overnight. For SEM, the cells treated with NZ2114 were dehydrated by a series of graded ethanol, dried by CO_2_, sputtered using platinum coating, and observed using SEM (QUANTA200, FEI, Philips, Amsterdam, The Netherlands). For TEM, the cells were post-fixed for 1 h with 1% OsO_4_, dehydrated by a graded acetone, and immersed in epoxy resin. Thin sections were cut using an ultramicrotome, followed by staining with 1% uranyl acetate. Images were observed using a TEM (JEM1400, JEOL, Tokyo, Japan) [53].

#### 4.3.2. Membrane Integrity Analysis by Flow Cytometry

To analyze the permeabilization of the bacteria membrane after NZ2114 treatment, the *S. pseudintermedius* cells (5 × 10^8^ cells/mL) were treated with 1× MIC NZ2114 at 37 °C for 30, 60, 90, and 120 min, respectively. The bacteria without NZ2114 or antibiotic treatment were used as a negative control and the bacteria treated with mupirocin, lincomysin, and nisin were used as positive controls, respectively. The cells were harvested by centrifugation, suspended with PBS (0.01 mM, pH 7.4), treated with 50 μg/mL PI at 37 °C for 15 min, and determined using a flow cytometer (FACS Calibur, BD, USA, Franklin Lake) [50].

#### 4.3.3. Super-Resolution Microscopy (SRM) Observation

The overnight-activated bacteria were transferred and grown to logarithmic growth phase, and the bacterial cells were diluted with PBS to 5 × 10^8^ CFU/mL. The bacterial solution was incubated at a final concentration of 4× MIC FITC-NZ2114 peptide solution at 37 °C for 30 min in the dark, washed with PBS, and stained for 15 min with PI and DAPI (Sigma-Aldrich LLC., Shanghai, China) at 4 °C. After being concentrated 10-fold, the samples were placed on polyTM microscope slides, an anti-fluorescence quencher was added, and the slides were sealed with coverslips and observed with an SRM (N-SIMS, Nikon, Japan) [53].

#### 4.3.4. Preparation of Lipids

Two types of liposomes were prepared to mimic the Gram-positive membrane (mass ratio of PG:CL= 3:1) and Gram-negative membrane (mass ratio of PG:CL:PE = 2:1:7). The phospholipids were dissolved with chloroform according to the ratio previously described and dried. The solvent was removed by spinning, and the residual organic solvent was removed by drying overnight in a vacuum desiccator [54,55]. LUVs’ encapsulated calcein was prepared according to the freeze–thaw method. A dye solution (10 mM calcein) was added into the samples. LUVs were prepared in liquid nitrogen via ten freeze–thaw cycles, and incubated in a 50 °C water bath. The suspensions were extruded through 200 nm polycarbonate membranes ten times. The unbound calcein was removed by 6–7 centrifugations (10000 rpm, 10 min). The volume was adjusted to 1 mL using the buffer (50 mM Tris-HCl, 100 mM NaCl, pH = 7.4), followed by washing to obtain a supernatant free of fluorescent dye. Finally, the calcein-loading LUVs were stored in dark conditions at 4 °C for leakage measurements.

#### 4.3.5. Calcein Leakage Assay

A 90 μL sample of LUVs and 10 μL of NZ2114 (or 10% Triton X-100) were added into a black 96-well plate, and the reaction mixture was plated away from light for 30 min. The calcein released from the LUVs was recorded by fluorescence intensity with the excitation wavelength of 470 nm and the emission wavelength of 520 nm. The disruption of liposomes by the peptide was assessed by analyzing the fluorescence values at different concentrations of peptide [54,55]. The percentage of released calcein was calculated using the following equation: Release (%) = 100 (F_t_ − F_0_)/(F_max_ − F_0_), where the fluorescence intensity before and after the addition of peptides is represented by F_0_ and F_t_, respectively, and F_max_ is the fluorescence intensity following Triton X-100 addition.

#### 4.3.6. Fluorescence Detection of Intracellular ROS Activity

The mid-log phase *S. pseudintermedius* cells (1 × 10^8^ CFU/mL) were added to DCFH-DA (final concentration: 10 μM), cultured at 37 °C for 30 min in the dark, and washed with PBS three times. The bacteria with fluorescent probes and peptides (final concentrations: 1×, 2× or 4× MIC) were added to a black 96-well plate, and incubated at 37 °C for 30 min. The fluorescence intensity of the samples was detected using a microplate reader (excitation wavelength of 488 nm and emission wavelength of 525 nm) [56].

#### 4.3.7. Alamar Blue Detection Cell Metabolic Activity

The *S. pseudintermedius* cells in mid-log phase were diluted with PBS to 1 × 10^6^ CFU/mL, and then inoculated into black 96-well plates. The peptides were added at final concentrations of 1×, 2×, or 4× MIC, and cultured for 6 h at 200 rpm and 37 °C. A 10 μL sample of resazurin (50 μg/mL) was added and incubated at 37 °C for 1 h in the dark, and the biofilm metabolic activity was detected at 571 nm using an enzyme meter [57].

### 4.4. Effect of NZ2114 on Biofilm

#### 4.4.1. Effect of NZ2114 on Inhibit Biofilm Formation

The biofilm-forming capacity of *S. pseudintermedius* was measured by crystal violet staining [46]. The mid-log phase *S. pseudintermedius* cells were diluted with the TSB (BEIJING AOBOXING BIO-TECH Co., Ltd.) broth to 1 × 10^8^ CFU/mL, mixed with peptides in 96-well plates at final concentrations varying from 0.5 to 16× MIC and incubated at 37 °C for 24 h. After washing with PBS 3 times to remove the planktonic cells, the biofilms were stained for 30 min with 0.1% crystal violet, washed with PBS, dried, and dissolved in 95% ethanol. The absorbance was detected at 570 nm using a microplate reader [15]. The untreated bacteria served as a blank control.

#### 4.4.2. Biofilm Observation by CLSM

The biofilms were observed by CLSM to further investigate the impact of NZ2114 on both the biofilm and internal bacteria. The bacteria in the mid-log phase were diluted with TSB broth to 1 × 10^8^ CFU/mL. The bacterial suspension (1 mL) was added to the 35 mm Petri dish, and co-incubated with peptides or antibiotics at a final concentration of 4× MIC for 24 h. After co-incubation, the biofilms were washed with PBS 3 times and then dyed for 15 min with PI and SYTO 9. After washing with PBS, the slides were observed using CLSM (LSM880, Zeiss, Oberkochen, Germany). The excitation/emission wavelengths for SYTO 9 stain are 480/500 nm, and those for PI are 490/635 nm. The group without peptides or antibiotic treatment was the control group [15].

### 4.5. Mouse In Vivo Test

The mouse pyoderma model was established to assess the in vivo efficacy of NZ2114. Six-week-old female BALB/c mice were randomly allocated into four groups, which included a blank control group (uninfected and injected with PBS), negative control group (infected and injected with PBS), and two treatment groups (infected and injected with NZ2114 or lincomycin). There were 20 mice in each group. All of the mice were injected subcutaneously with 100 μL of *S. pseudintermedius* CGMCC 1.90024 (8 × 10^8^ CFU/mL). After *S. pseudintermedius* infection for 4 h, mice were injected intraperitoneally with NZ2114 or lincomycin (5 mg/kg) for a total of three treatments, each 12 h apart. The abdominal infection sites of the mice were photographed and the area measured by Image J on days 3, 7, and 14 after treatment. The mice were euthanized, and their skin tissues were collected, weighed, and homogenized. The homogenized samples were serially diluted with sterile PBS for colony counting. At the same time, the skin samples from mice on days 7 and 14 after treatment were fixed overnight in 4% paraformaldehyde, stained with hematoxylin and eosin, and observed using a light microscope (BX43, OLYMPUS) [21]. The mouse experiment was performed according to the Animal Care and Use Committee of the Feed Research Institute of the Chinese Academy of Agricultural Sciences (CAAS) and approved by the Laboratory Animal Ethical Committee and its Inspection of the Feed Research Institute of CAAS (IFR-CAAS20230615).

### 4.6. Statistical Analysis

All experiments were independently repeated three times. Data are shown as mean ± SEM and the data were statistically analyzed by one-way ANOVA. All evaluations were performed using GraphPad Prism version 8.0, and the results were considered significant at *p* < 0.05 and extremely significant at *p* < 0.001.

## 5. Conclusions

In this study, the in vitro and in vivo antibacterial activity and mechanisms of action of NZ2114 against *S. pseudintermedius* were systematically investigated. NZ2114 exhibited potent antibacterial activity with a lower probability of inducing drug-resistant mutations and efficient bactericidal action. NZ2114 possessed the dual mechanism involving both destruction of the cell membrane and interference of intracellular metabolic activities. NZ2114 also effectively inhibited biofilm formation, eliminating biofilm and its internal bacteria. The in vivo therapy of NZ2114 in a mouse pyoderma model induced by *S. pseudintermedius* showed that it effectively decreased the number of skin bacteria, alleviated histological damage, and reduced the skin damage area. These results, including efficacy results on preparation optimization together [19], fully demonstrated that NZ2114 can potentially be used in the treatment of *S. pseudintermedius* infections.

## Figures and Tables

**Table 1:** MIC and MBC values of NZ2114 and antibiotics.

Strains	NZ2114				Mupirocin				Lincomycin			
	MIC		MBC		MIC		MBC		MIC		MBC	
	μg/mL	μM	μg/mL	μM	μg/mL	μM	μg/mL	μM	μg/mL	μM	μg/mL	μM
*S. pseudintermedius* CGMCC 1.90024	1	0.23	2	0.46	0.25	0.5	16	32	32	69.41	64	138.83
*S. pseudintermedius* CGMCC 1.90005	1	0.23	1	0.23	0.125	0.25	0.5	1	2	4.34	>256	>555.30

## Data Availability

The original contributions presented in the study are included in the article; further inquiries can be directed to the corresponding authors.
